# Supplementary material for: Diet, Lifestyle Factors, and Quality of Life in Patients with Rheumatic Diseases: A Cross-Sectional Study
Source: Nutrients. 2025 Nov 7;17(22):3499. doi: 10.3390/nu17223499 (PMC12655434; doi:10.3390/nu17223499)
Supplement: Supplementary file 1 [file nutrients-17-03499-s001.zip › nutrients-3965226-supplementary.pdf]

Questionnaire: Nutrition and Lifestyle in Patients with Rheumatic Conditions

Hello,

You are invited to participate in a research study conducted by the research team at the Doctoral School of Biomedical Sciences, "Dunărea de Jos" University of Galați, Romania. This study is part of an ongoing doctoral research project coordinated by Verga (Răuță) Gabriela Isabela, a third-year PhD student, under the supervision of academic and clinical experts in rheumatology and public health.

The aim of the study is to evaluate the relationship between dietary habits, lifestyle behaviors, and self-perceived health status among individuals diagnosed with rheumatic conditions.

Completing the questionnaire will take approximately 15–20 min.

By proceeding, you are providing your informed consent to voluntarily participate in this study.

All responses are anonymous and confidential, and the data collected will be used exclusively for scientific and statistical purposes. Your personal information will be processed in accordance with Law no. 677/2001 and EU General Data Protection Regulation (GDPR) 2016/679.

We sincerely thank you for your time and valuable contribution to this research.

Section 1. General Information

1. **What is your age?**

- ☐ 18–25 years
- ☐ 26–35 years
- ☐ 36–45 years
- ☐ 46–55 years
- ☐ Over 55 years

2. **Your gender:**

- ☐ Male
- ☐ Female

3. **Your place of residence:**

- ☐ Urban
- ☐ Rural

4. **Your highest level of education completed:**

- ☐ Primary education
- ☐ Secondary education (high school)
- ☐ Post-secondary / vocational studies
- ☐ Bachelor's degree
- ☐ Master's degree
- ☐ Doctoral degree

5. **What is your current occupational status?**

- ☐ Unemployed
- ☐ Homemaker
- ☐ Jobseeker
- ☐ Freelancer
- ☐ Student
- ☐ Employed
- ☐ Manager
- ☐ Retired

6. **Are you registered as having a disability due to your rheumatic condition?**

- ☐ Yes
- ☐ No

7. **What percentage of your monthly income do you spend on food?**

- ☐ 10–25%
- ☐ 26–50%
- ☐ 51–70%
- ☐ Over 70%

8. **Your height:**

- ☐ 140–150 cm
- ☐ 150–160 cm
- ☐ 160–170 cm
- ☐ 170–180 cm
- ☐ 180–190 cm
- ☐ 190–200 cm
- ☐ Over 200 cm

9. **Your weight:**

- ☐ 40–50 kg
- ☐ 50–60 kg
- ☐ 60–70 kg
- ☐ 70–80 kg
- ☐ 80–90 kg
- ☐ 90–100 kg
- ☐ Over 100 kg

Section 2. Rheumatic Diagnosis and Treatment

10. **What is your rheumatic diagnosis?** *(You may select more than one)*

- ☐ Rheumatoid arthritis
- ☐ Ankylosing spondylitis
- ☐ Systemic lupus erythematosus
- ☐ Sjögren's syndrome
- ☐ Vasculitis
- ☐ Osteoarthritis
- ☐ Gout
- ☐ Polymyalgia rheumatica
- ☐ Scleroderma

11. **How long have you had this diagnosis?**

- ☐ Less than 1 year
- ☐ 1–2 years
- ☐ 3–5 years
- ☐ 6–8 years
- ☐ 9–11 years
- ☐ 12–15 years
- ☐ 16–20 years
- ☐ Over 20 years

12. **Are you currently under the care of a rheumatologist?**

- ☐ Yes
- ☐ No

13. **What type of treatment are you currently following for your rheumatic condition?** *(Select all that apply)*

- ☐ Non-steroidal anti-inflammatory drugs (NSAIDs)
- ☐ Corticosteroids
- ☐ Disease-modifying antirheumatic drugs (DMARDs—e.g., Methotrexate, Sulfasalazine)
- ☐ Biologics (e.g., Infliximab, Adalimumab, Etanercept)
- ☐ Painkillers/Analgesics
- ☐ Supplements (e.g., vitamin D, calcium)

14. **How long have you been on your current treatment plan?**

- ☐ Less than 6 months
- ☐ 6–12 months
- ☐ 1–3 years
- ☐ More than 3 years

15. **Have you tried any complementary therapies?** *(Tick what applies)*

- ☐ No
- ☐ Physiotherapy
- ☐ Acupuncture
- ☐ Homeopathy

16. **Have you had any surgical interventions due to your rheumatic condition?**

- ☐ Yes
- ☐ No

17. **To what extent do you believe your medication has affected your body weight?**

- ☐ To a very great extent
- ☐ To a great extent
- ☐ Neutral
- ☐ To a small extent
- ☐ To a very small extent
- ☐ Not at all

18. **Do you take your medication regularly as prescribed by your doctor?**

- ☐ Yes
- ☐ No
- ☐ Occasionally

19. **Have you experienced any significant side effects from your treatment?**

- ☐ Yes
- ☐ No

Section 3. Eating Habits and Lifestyle

20. **Do you have any known food intolerances?**

- ☐ Yes
- ☐ No

21. **How many meals do you usually have per day?**

- ☐ 1 meal
- ☐ 2 meals
- ☐ 3 meals
- ☐ 3 meals with 2 snacks

22. **How often do you eat fast food?**

- ☐ Daily

- Twice a week
- Weekly
- Monthly
- Rarely/Never

23. **How often do you consume home-cooked meals?**

- Daily
- Twice a week
- Weekly
- Monthly
- Rarely/Never

24. **How often do you eat chicken?**

- Daily
- Twice a week
- Weekly
- Monthly
- Rarely/Never

25. **How often do you eat beef?**

- Daily
- Twice a week
- Weekly
- Monthly
- Rarely/Never

26. **How often do you consume the following per week?**

Product	Daily	Twice a Week	Weekly	Monthly	Rarely/Never
Fish	[ ]	[ ]	[ ]	[ ]	[ ]
Seafood	[ ]	[ ]	[ ]	[ ]	[ ]

27. **What type of milk do you consume most often?**

- Whole cow's milk
- Semi-skimmed/skimmed cow's milk
- Lactose-free milk
- Plant-based milk (soy, almond, oat, coconut, etc.)
- Goat's milk
- I do not drink milk

28. **What type of fats do you usually use for cooking?**

- Vegetable oil (sunflower, corn)
- Olive oil
- Butter
- Margarine
- Lard/animal fat
- Coconut/palm oil
- I do not cook
- Other oils (e.g., rapeseed, sesame, flaxseed, pumpkin, grape seed, avocado, walnut)

29. **How much water do you drink daily?**

- 0–1 liter
- 1–2 liters
- 2–3 liters
- 3–4 liters
- I do not drink water

- I don’t know

30. **What is your preferred method of food preparation?** *(select up to 2 options)*

- Boiling
- Baking
- Grilling
- Air frying
- Shallow frying
- Deep frying
- Sautéing/light frying
- Steaming
- Raw/salads
- I do not cook

Section 4. Perception of Diet

31. **Did you change your diet after being diagnosed with your rheumatic condition?**

- Yes, in terms of quantity
- Yes, in terms of quality
- No

32. **Which of the following foods do you consider beneficial for your health?**

(Tick your level of agreement)

Food Categories	Strongly Agree	Agree	Neutral	Disagree	Strongly Disagree
Vegetables and leafy greens	[ ]	[ ]	[ ]	[ ]	[ ]
Fruits	[ ]	[ ]	[ ]	[ ]	[ ]
Whole grains	[ ]	[ ]	[ ]	[ ]	[ ]
Legumes	[ ]	[ ]	[ ]	[ ]	[ ]
Dairy and alternatives	[ ]	[ ]	[ ]	[ ]	[ ]
Lean animal proteins	[ ]	[ ]	[ ]	[ ]	[ ]
Nuts and seeds	[ ]	[ ]	[ ]	[ ]	[ ]
Healthy fats	[ ]	[ ]	[ ]	[ ]	[ ]

33. **Which of the following foods have worsened your symptoms?**

(Tick your level of agreement)

Food Items	Strongly Agree	Agree	Neutral	Disagree	Strongly Disagree
Processed and red meat	[ ]	[ ]	[ ]	[ ]	[ ]
Spicy/heavily seasoned foods	[ ]	[ ]	[ ]	[ ]	[ ]
Dairy products	[ ]	[ ]	[ ]	[ ]	[ ]
Refined carbohydrates	[ ]	[ ]	[ ]	[ ]	[ ]
Gluten-containing foods	[ ]	[ ]	[ ]	[ ]	[ ]
Sweets and sugary products	[ ]	[ ]	[ ]	[ ]	[ ]
Fizzy/sugary drinks	[ ]	[ ]	[ ]	[ ]	[ ]
Alcohol	[ ]	[ ]	[ ]	[ ]	[ ]
Coffee/caffeinated beverages	[ ]	[ ]	[ ]	[ ]	[ ]
Refined foods	[ ]	[ ]	[ ]	[ ]	[ ]

34. **To what extent have you noticed pain relief as a result of dietary changes?**

- To a very great extent
- To a great extent
- Neutral
- To a small extent
- To a very small extent

35. **To what extent do you believe diet influences the symptoms of your condition?**

- To a very great extent
- To a great extent
- Neutral
- To a small extent
- To a very small extent

36. **How often do you consume alcohol?**

- Very often
- Often
- Neutral
- Rarely
- Very rarely
- Never

37. **How often do you smoke?**

- Very often
- Often
- Neutral
- Rarely
- Very rarely
- Never

38. **Do you follow a special diet?**

- Yes
- No

Section 5. Quality of Life Self-Assessment

39. **How much do you think rheumatic disease affects your quality of life, on a scale of 1 to 10?**

(1 = very poor, 10 = very good)

[ ] 1 [ ] 2 [ ] 3 [ ] 4 [ ] 5 [ ] 6 [ ] 7 [ ] 8 [ ] 9 [ ] 10

40. **How would you rate your quality of life at present?**

(1 = very poor, 10 = very good)

[ ] 1 [ ] 2 [ ] 3 [ ] 4 [ ] 5 [ ] 6 [ ] 7 [ ] 8 [ ] 9 [ ] 10

41. **Do you feel that you have control over your condition?**

- Yes
- No
- Partially

42. **On a scale from 1 to 10, to what extent have your social relationships been affected by your condition?**

(1 = very affected, 10 = not affected at all)

[ ] 1 [ ] 2 [ ] 3 [ ] 4 [ ] 5 [ ] 6 [ ] 7 [ ] 8 [ ] 9 [ ] 10

43. **On a scale from 1 to 10, to what extent has your emotional state been affected?**

(1 = very poor, 10 = very good)

[ ] 1 [ ] 2 [ ] 3 [ ] 4 [ ] 5 [ ] 6 [ ] 7 [ ] 8 [ ] 9 [ ] 10

44. **To what extent has your sleep been affected?**

- To a very great extent
- To a great extent
- Neutral

- To a small extent
- To a very small extent
- Not at all

45. **To what extent are your daily activities affected?**

- To a very great extent
- To a great extent
- Neutral
- To a small extent
- To a very small extent
- Not at all

46. **To what extent do you regularly engage in physical activity?**

**Type of Activity**	**Very Great Extent**	**Great Extent**	**Neutral**	**Small Extent**	**Very Small Extent**
Walking	[ ]	[ ]	[ ]	[ ]	[ ]
Running	[ ]	[ ]	[ ]	[ ]	[ ]
Sports	[ ]	[ ]	[ ]	[ ]	[ ]
Exercise	[ ]	[ ]	[ ]	[ ]	[ ]

47. **Which type of diet do you believe is most appropriate for your condition?**

(Tick your level of agreement)

**Diet Type**	**Strongly Agree**	**Agree**	**Neutral**	**Disagree**	**Strongly Disagree**
Low-calorie diet (for weight loss)	[ ]	[ ]	[ ]	[ ]	[ ]
Low-carb diet	[ ]	[ ]	[ ]	[ ]	[ ]
Low-fat diet	[ ]	[ ]	[ ]	[ ]	[ ]
Low-sodium diet	[ ]	[ ]	[ ]	[ ]	[ ]
High-fiber diet	[ ]	[ ]	[ ]	[ ]	[ ]
Low-protein diet	[ ]	[ ]	[ ]	[ ]	[ ]
Gluten-free diet	[ ]	[ ]	[ ]	[ ]	[ ]
Lactose-free diet	[ ]	[ ]	[ ]	[ ]	[ ]
Vegetarian/Vegan diet	[ ]	[ ]	[ ]	[ ]	[ ]
Mediterranean diet	[ ]	[ ]	[ ]	[ ]	[ ]
DASH diet (for hypertension)	[ ]	[ ]	[ ]	[ ]	[ ]

48. **On a scale from 1 to 10, to what extent does your diet influence your quality of life?**

(1 = not at all, 10 = extremely)

[ ] 1 [ ] 2 [ ] 3 [ ] 4 [ ] 5 [ ] 6 [ ] 7 [ ] 8 [ ] 9 [ ] 10
